# Tris(*N*,*N*-di­methyl­anilinium) tri-μ-bromido-bis[tribromido­anti­monate(III)]

**DOI:** 10.1107/S1600536813014335

**Published:** 2013-06-08

**Authors:** Houda Kharrat, Slaheddine Kamoun, François Michaud

**Affiliations:** a Laboratoire de Génie des Matériaux et Environnement, École Nationale d’Ingénieurs de Sfax, Université de Sfax, BP 1173, Sfax, Tunisia; b Service commun d’analyse par diffraction des rayons X, Universite de Brest, 6 avenue Victor Le Gorgeu, CS 93837, F-29238 Brest Cedex 3, France

## Abstract

In the title compound, (C_8_H_12_N)_3_[Sb_2_Br_9_], two of the three unique *N*,*N*-dimethyanilinium cations exhibit flip–flop disorder with an occupancy ratio of 0.58 (1):0.42 (1). N—H⋯Br hydrogen bonds link the organic cations and bioctahedral face-sharing anions into a three-dimensional network.

## Related literature

For related hybrid organic anti­monate(III) halogenide crystal structures, see: Bujak & Angel (2005 ▶); Chaabouni *et al.* (1997 ▶, 1998 ▶). For dielectric and phase transitions properties, see: Chaabouni & Kamoun (1998 ▶).
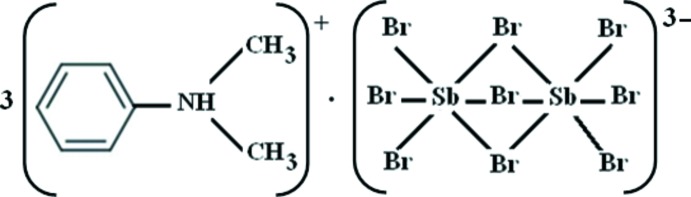


## Experimental

### 

#### Crystal data

(C_8_H_12_N)_3_[Sb_2_Br_9_]
*M* *_r_* = 1329.25
Triclinic, 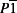

*a* = 9.7857 (3) Å
*b* = 13.7658 (5) Å
*c* = 17.0297 (6) Å
α = 66.581 (4)°
β = 78.689 (3)°
γ = 72.601 (3)°
*V* = 2000.91 (14) Å^3^
*Z* = 2
Mo *K*α radiation
μ = 10.36 mm^−1^
*T* = 296 K
0.58 × 0.29 × 0.20 mm

#### Data collection

Oxford Diffraction Xcalibur (Sapphire2) diffractometer
Absorption correction: multi-scan (*CrysAlis RED*; Oxford Diffraction, 2009 ▶) *T*_min_ = 0.038, *T*_max_ = 0.126
29905 measured reflections
12050 independent reflections
6232 reflections with *I* > 2σ(*I*)
*R*_int_ = 0.055

#### Refinement

*R*[*F* ^2^ > 2σ(*F* ^2^)] = 0.042
*wR*(*F* ^2^) = 0.096
*S* = 0.88
12050 reflections
398 parameters
88 restraints
H-atom parameters constrained
Δρ_max_ = 1.33 e Å^−3^
Δρ_min_ = −0.89 e Å^−3^

### 

Data collection: *CrysAlis CCD* (Oxford Diffraction, 2009 ▶); cell refinement: *CrysAlis RED* (Oxford Diffraction, 2009 ▶); data reduction: *CrysAlis RED*; program(s) used to solve structure: *SIR92* (Altomare *et al.*, 1993 ▶); program(s) used to refine structure: *SHELXL97* (Sheldrick, 2008 ▶); molecular graphics: *DIAMOND* (Brandenburg & Berndt, 1999 ▶) and *Mercury* (Macrae *et al.*, 2008 ▶); software used to prepare material for publication: *WinGX* (Farrugia, 2012 ▶) and *publCIF* (Westrip, 2010 ▶).

## Supplementary Material

Crystal structure: contains datablock(s) global, I. DOI: 10.1107/S1600536813014335/vn2071sup1.cif

Supplementary material file. DOI: 10.1107/S1600536813014335/vn2071Isup2.cdx

Structure factors: contains datablock(s) I. DOI: 10.1107/S1600536813014335/vn2071Isup3.hkl

Additional supplementary materials:  crystallographic information; 3D view; checkCIF report

## Figures and Tables

**Table 1:** Selected bond lengths (Å)

Sb1—Br1	2.6036 (6)
Sb1—Br2	2.6292 (6)
Sb1—Br3	2.6925 (6)
Sb1—Br4	3.1222 (6)
Sb1—Br5	3.0287 (6)
Sb1—Br6	2.9558 (7)
Sb2—Br4	3.1333 (7)
Sb2—Br5	3.1602 (7)
Sb2—Br6	3.2393 (7)
Sb2—Br7	2.6068 (7)
Sb2—Br8	2.5696 (7)
Sb2—Br9	2.5713 (8)

**Table 2:** Hydrogen-bond geometry (Å, °)

*D*—H⋯*A*	*D*—H	H⋯*A*	*D*⋯*A*	*D*—H⋯*A*
N1—H1⋯Br4	0.91	2.47	3.362 (5)	168
N2*A*—H2*A*⋯Br3	0.91	2.43	3.23 (3)	146
N2*B*—H2*B*⋯Br3	0.91	2.55	3.43 (4)	162
N3*A*—H3*A*⋯Br6	0.91	2.4	3.25 (2)	155
N3*B*—H3*B*⋯Br6	0.91	2.68	3.48 (3)	148

**Fig. 1.**
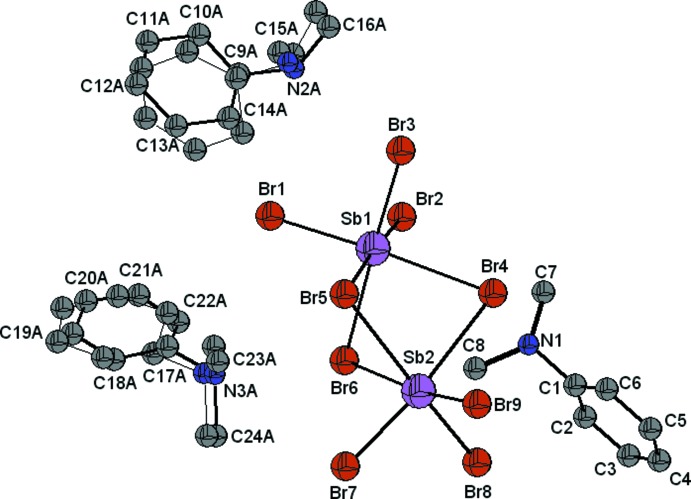
The asymmetric part of the unit cell of (C8H12N)3Sb2Br9 crystal at 296 (2) K with the atom labeling scheme.

**Fig. 2.**
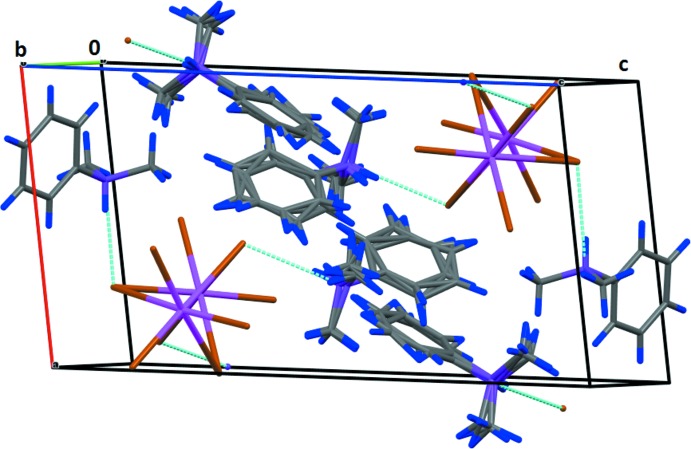
The crystal packing of (C8H12N)3Sb2Br9 showing the hydrogen bonding network as blue dashed lines.

## supplementary crystallographic information

### Comment

This study is a part of our investigations on the syntheses, structures and phase transitions of halogenoantimonate (III) anions in combination with organic cations (Bujak & Angel, 2005; Chaabouni *et al.*, 1997; Chaabouni *et al.*, 1998; Chaabouni & Kamoun, 1998). In these compounds the Sb atom shows a tendency toward distorted octahedral coordination with some rather long Sb—*X* bonds, which is attributed to the aspherical distribution of the lone pair electrons at Sb(III). The asymmetric unit of the title compound, consists of one isolated [Sb_2_Br_9_]^3-^ anion with one ordered and two disordered *N*,*N*-dimethylanilinium cations which exhibit flip-flop disorder with 0.58 (1):0.42 (1) occupancy ratio. The inorganic ions are built up from two distorted [SbBr_6_]^3-^ octahedra sharing one face. Two types of Sb—Br distances are present within the Sb_2_Br_9_ group: six short Sb—Br (terminal) distances [2.5696 (7)–2.6925 (6) Å)] and three longer Sb—Br (bridging) distances [(2.9558 (7)–3.2393 (7) Å)]. The interatomic Br—Sb—Br angles involving the terminal Br atoms are greater than the ideal 90°, whereas those between the bridging ones are less than 90°. The average Sb—Br—Sb angle is 89.92 (2)°. According to the valence-shell electron-pair repulsion (VSEPR) model, the lone pair electrons on Sb(III) atom may be considered as stereochemical non active. The phenyl rings of the *N*,*N*-dimethylanilinium cations are practically planar with the greatest deviation from the six-atoms least squares plane being 0.0088 Å, 0.0383 Å,0.0518 Å, 0.0605 Å and 0.0970 Å respectively. The π-π interactions between phenyl rings may be neglected (> 4 Å); in fact the shortest distances between the centroids of the rings are: *Cg*(1)···*Cg*(1)^i^ = 5.521 (4) Å,*Cg*(2)···*Cg*(2)^ii^ = 4.227 (15) Å, *Cg*(2)···*Cg*(3)^ii^ = 4.214 (19) Å and *Cg*(3)···*Cg*(3)^ii^ = 4.26 (2) Å [*Cg*(1), *Cg*(2) and *Cg*(3) are the centroids of the C1···C6, C9···C14 and C17···C22 rings respectively; symmetry codes: (i) 1 - *x*, -*y*, -*z*; (ii) -*x*, 1 - *y*, 1 - *z*]. The major contributions to the cohesion and the stability of the structure is the presence of N—H···Br hydrogen bonds which provide a linkage between *N*,*N*-dimethylanilinium cations and [Sb_2_Br_9_]^3-^ anions which include five relatively medium contacts, with H···Br and N..Br distances ranging from 2.40 Å to 2.68 Å and 3.23 (3) Å to 3.48 (3) Å, respectively (Fig. 2 and Table 1).

### Experimental

Crystals of (C_8_H_12_N)_3_[Sb_2_Br_9_] were obtained by dissolving antimony (III) oxide Sb_2_O_3_ and *N*,*N*-dimethylanilinium bromide C_8_H_12_NBr (molar ratio 3:1) in 50 ml of a solution of HBr (24%). After a slow solvent evaporation prismatic yellow crystals suitable for X-ray analysis were obtained. They were washed with diethyl ether and dried over P_2_O_5_.

### Refinement

The positions of the two disordered *N*,*N*-dimethylanilinium cations were initially refined with different occupancy ratios, but the refinement converged extremely slowly when using anisotropic atomic displacement parameters. Since the refined occupancy factors for the two cations were nearly equal upon refining with isotropic atomic displacement parameters it was decided to use a single occupancy factor for the final anisotropic refinement. SADI and EADP restraints were used. All H atoms were geometrically positioned and treated as riding on their parent atoms, with C—H = 0.93 Å for the phenyl, 0.96 Å for the methyl and N—H = 0.91 Å with *U*_iso_(H) = 1.2 *U*_eq_(*C*-phenyl, N) or 1.5 *U*_eq_(*C*-methyl).

### Crystal data

**Table:**

(C_8_H_12_N)_3_[Sb_2_Br_9_]	*Z* = 2
*M**_r_* = 1329.25	*F*(000) = 1236
Triclinic, *P*1	*D*_x_ = 2.206 Mg m^−^^3^
Hall symbol: -P 1	Mo *K*α radiation, λ = 0.71073 Å
*a* = 9.7857 (3) Å	Cell parameters from 8586 reflections
*b* = 13.7658 (5) Å	θ = 3.1–44.7°
*c* = 17.0297 (6) Å	µ = 10.36 mm^−^^1^
α = 66.581 (4)°	*T* = 296 K
β = 78.689 (3)°	Truncated prism, axis [1 0 0], light yellow
γ = 72.601 (3)°	0.58 × 0.29 × 0.20 mm
*V* = 2000.91 (14) Å^3^	

### Data collection

**Table:**

Oxford Diffraction Xcalibur (Sapphire2, large Be window) diffractometer	12050 independent reflections
Radiation source: sealed X-ray tube	6232 reflections with *I* > 2σ(*I*)
Graphite monochromator	*R*_int_ = 0.055
Detector resolution: 8.3622 pixels mm^-1^	θ_max_ = 30.5°, θ_min_ = 3.1°
ω and π scans	*h* = −13→12
Absorption correction: multi-scan (*CrysAlis RED*; Oxford Diffraction, 2009)	*k* = −19→19
*T*_min_ = 0.038, *T*_max_ = 0.126	*l* = −23→24
29905 measured reflections	

### Refinement

**Table:**

Refinement on *F*^2^	Primary atom site location: structure-invariant direct methods
Least-squares matrix: full	Secondary atom site location: difference Fourier map
*R*[*F*^2^ > 2σ(*F*^2^)] = 0.042	Hydrogen site location: inferred from neighbouring sites
*wR*(*F*^2^) = 0.096	H-atom parameters constrained
*S* = 0.88	*w* = 1/[σ^2^(*F*_o_^2^) + (0.0451*P*)^2^] where *P* = (*F*_o_^2^ + 2*F*_c_^2^)/3
12050 reflections	(Δ/σ)_max_ = 0.001
398 parameters	Δρ_max_ = 1.33 e Å^−^^3^
88 restraints	Δρ_min_ = −0.89 e Å^−^^3^

### Special details

**Table:**

Experimental. Absorption correction: empirical absorption correction using spherical harmonics, implemented in SCALE3 ABSPACK scaling algorithm. CrysAlis RED (Oxford Diffraction 2009)
Geometry. All s.u.'s (except the s.u. in the dihedral angle between two l.s. planes) are estimated using the full covariance matrix. The cell s.u.'s are taken into account individually in the estimation of s.u.'s in distances, angles and torsion angles; correlations between s.u.'s in cell parameters are only used when they are defined by crystal symmetry. An approximate (isotropic) treatment of cell s.u.'s is used for estimating s.u.'s involving l.s. planes.
Refinement. Refinement of *F*^2^ against ALL reflections. The weighted *R*-factor *wR* and goodness of fit *S* are based on *F*^2^, conventional *R*-factors *R* are based on *F*, with *F* set to zero for negative *F*^2^. The threshold expression of *F*^2^ > 2σ(*F*^2^) is used only for calculating *R*-factors(gt) *etc*. and is not relevant to the choice of reflections for refinement. *R*-factors based on *F*^2^ are statistically about twice as large as those based on *F*, and *R*- factors based on ALL data will be even larger.

### Fractional atomic coordinates and isotropic or equivalent isotropic displacement parameters (Å^2^)

**Table:**

	*x*	*y*	*z*	*U*_iso_*/*U*_eq_	Occ. (<1)
Sb1	0.74142 (3)	0.34793 (2)	0.23720 (2)	0.03514 (9)	
Sb2	0.79395 (4)	0.05119 (3)	0.24531 (2)	0.04300 (10)	
Br1	0.75743 (7)	0.40023 (5)	0.36561 (4)	0.05958 (17)	
Br2	0.50427 (6)	0.50174 (4)	0.19319 (4)	0.05655 (16)	
Br3	0.91249 (6)	0.47755 (5)	0.13260 (4)	0.05430 (15)	
Br4	0.73112 (6)	0.27216 (5)	0.08874 (4)	0.05175 (14)	
Br5	1.00278 (6)	0.15806 (5)	0.28605 (4)	0.05553 (15)	
Br6	0.56880 (6)	0.19270 (5)	0.35018 (4)	0.05216 (15)	
Br7	0.83710 (9)	−0.11714 (5)	0.38803 (4)	0.0767 (2)	
Br8	0.59360 (7)	−0.00822 (6)	0.21190 (4)	0.06810 (18)	
Br9	0.98781 (8)	−0.04384 (5)	0.15561 (5)	0.0780 (2)	
C1	0.3358 (6)	0.2456 (4)	0.0464 (3)	0.0491 (14)	
C2	0.1982 (7)	0.2361 (5)	0.0576 (4)	0.0615 (16)	
H2	0.1274	0.2694	0.0905	0.074*	
C3	0.1669 (8)	0.1756 (6)	0.0186 (4)	0.0719 (19)	
H3	0.0737	0.1678	0.0253	0.086*	
C4	0.2687 (10)	0.1279 (6)	−0.0287 (5)	0.079 (2)	
H4	0.2453	0.0859	−0.053	0.094*	
C5	0.4050 (10)	0.1392 (6)	−0.0422 (5)	0.088 (2)	
H5	0.4741	0.1071	−0.0765	0.106*	
C6	0.4396 (7)	0.2009 (6)	−0.0030 (4)	0.0730 (19)	
H6	0.5321	0.2106	−0.0111	0.088*	
C7	0.3312 (7)	0.4277 (4)	0.0415 (4)	0.0618 (16)	
H7A	0.3603	0.4649	0.0701	0.093*	
H7B	0.2286	0.45	0.0403	0.093*	
H7C	0.376	0.4455	−0.0162	0.093*	
C8	0.3250 (7)	0.2778 (5)	0.1818 (3)	0.0626 (16)	
H8A	0.3555	0.3201	0.2052	0.094*	
H8B	0.3648	0.2015	0.2117	0.094*	
H8C	0.222	0.2926	0.1885	0.094*	
N1	0.3760 (5)	0.3075 (4)	0.0885 (3)	0.0548 (12)	
H1	0.4738	0.2894	0.0854	0.066*	
C9A	1.062 (5)	0.5602 (16)	0.316 (2)	0.066 (4)	0.577 (12)
C10A	1.085 (4)	0.6280 (15)	0.3488 (14)	0.083 (4)	0.577 (12)
H10A	1.0581	0.7031	0.3198	0.099*	0.577 (12)
C11A	1.148 (3)	0.5882 (13)	0.4232 (15)	0.110 (6)	0.577 (12)
H11A	1.149	0.6346	0.4505	0.132*	0.577 (12)
C12A	1.209 (2)	0.4806 (15)	0.4572 (13)	0.096 (6)	0.577 (12)
H12A	1.258	0.4556	0.5058	0.116*	0.577 (12)
C13A	1.203 (3)	0.4034 (17)	0.4235 (14)	0.104 (7)	0.577 (12)
H13A	1.2433	0.329	0.449	0.125*	0.577 (12)
C14A	1.131 (2)	0.4483 (15)	0.3484 (14)	0.066 (3)	0.577 (12)
H14A	1.129	0.4037	0.3197	0.079*	0.577 (12)
C15A	0.849 (3)	0.684 (5)	0.240 (2)	0.074 (7)	0.577 (12)
H15A	0.8082	0.7135	0.1862	0.111*	0.577 (12)
H15B	0.864	0.7426	0.2523	0.111*	0.577 (12)
H15C	0.7834	0.649	0.285	0.111*	0.577 (12)
C16A	1.086 (4)	0.651 (2)	0.1585 (19)	0.065 (4)	0.577 (12)
H16A	1.0332	0.6842	0.1086	0.097*	0.577 (12)
H16B	1.1673	0.595	0.1511	0.097*	0.577 (12)
H16C	1.1187	0.706	0.1662	0.097*	0.577 (12)
N2A	0.990 (4)	0.603 (3)	0.2367 (18)	0.070 (3)	0.577 (12)
H2A	0.9706	0.5452	0.2309	0.084*	0.577 (12)
C9B	1.083 (7)	0.545 (2)	0.319 (3)	0.066 (4)	0.423 (12)
C10B	1.101 (5)	0.588 (2)	0.374 (2)	0.083 (4)	0.423 (12)
H10B	1.0614	0.6615	0.3642	0.099*	0.423 (12)
C11B	1.176 (5)	0.524 (2)	0.444 (2)	0.110 (6)	0.423 (12)
H11B	1.2036	0.5545	0.4759	0.132*	0.423 (12)
C12B	1.208 (4)	0.415 (2)	0.464 (2)	0.096 (6)	0.423 (12)
H12B	1.2545	0.3691	0.5132	0.116*	0.423 (12)
C13B	1.172 (4)	0.369 (2)	0.412 (2)	0.104 (7)	0.423 (12)
H13B	1.1997	0.2933	0.4274	0.125*	0.423 (12)
C14B	1.097 (3)	0.433 (2)	0.339 (2)	0.066 (3)	0.423 (12)
H14B	1.0619	0.4052	0.3076	0.079*	0.423 (12)
C15B	0.863 (5)	0.681 (7)	0.258 (4)	0.074 (7)	0.423 (12)
H15D	0.8078	0.709	0.2098	0.111*	0.423 (12)
H15E	0.8787	0.7402	0.2686	0.111*	0.423 (12)
H15F	0.8113	0.639	0.3082	0.111*	0.423 (12)
C16B	1.096 (5)	0.675 (3)	0.169 (3)	0.065 (4)	0.423 (12)
H16D	1.0522	0.7028	0.1164	0.097*	0.423 (12)
H16E	1.1905	0.6293	0.1633	0.097*	0.423 (12)
H16F	1.1037	0.7345	0.1825	0.097*	0.423 (12)
N2B	1.006 (5)	0.608 (4)	0.241 (2)	0.070 (3)	0.423 (12)
H2B	0.9876	0.5599	0.2226	0.084*	0.423 (12)
C17A	0.653 (3)	0.177 (2)	0.5912 (13)	0.049 (4)	0.577 (12)
C18A	0.6882 (18)	0.137 (3)	0.6726 (16)	0.060 (4)	0.577 (12)
H18A	0.7405	0.0648	0.6959	0.072*	0.577 (12)
C19A	0.648 (4)	0.200 (3)	0.721 (2)	0.086 (6)	0.577 (12)
H19A	0.6819	0.1759	0.7747	0.103*	0.577 (12)
C20A	0.559 (3)	0.299 (2)	0.6888 (13)	0.091 (7)	0.577 (12)
H20A	0.5361	0.3454	0.7198	0.109*	0.577 (12)
C21A	0.501 (4)	0.335 (4)	0.614 (2)	0.109 (7)	0.577 (12)
H21A	0.434	0.4021	0.594	0.13*	0.577 (12)
C22A	0.545 (3)	0.268 (3)	0.569 (3)	0.076 (6)	0.577 (12)
H22A	0.4995	0.285	0.5203	0.091*	0.577 (12)
C23A	0.848 (3)	0.0786 (13)	0.5141 (16)	0.043 (3)	0.577 (12)
H23A	0.8667	0.0384	0.4766	0.065*	0.577 (12)
H23B	0.8948	0.0337	0.5657	0.065*	0.577 (12)
H23C	0.8841	0.1429	0.4858	0.065*	0.577 (12)
C24A	0.624 (4)	0.016 (3)	0.572 (3)	0.065 (8)	0.577 (12)
H24A	0.6498	−0.0241	0.5339	0.098*	0.577 (12)
H24B	0.521	0.0419	0.5778	0.098*	0.577 (12)
H24C	0.657	−0.031	0.6273	0.098*	0.577 (12)
N3A	0.691 (2)	0.111 (2)	0.5359 (16)	0.066 (3)	0.577 (12)
H3A	0.6518	0.155	0.4855	0.079*	0.577 (12)
C17B	0.646 (4)	0.166 (3)	0.615 (2)	0.049 (4)	0.423 (12)
C18B	0.728 (3)	0.124 (4)	0.685 (3)	0.060 (4)	0.423 (12)
H18B	0.8106	0.0671	0.6913	0.072*	0.423 (12)
C19B	0.672 (6)	0.175 (4)	0.745 (3)	0.086 (6)	0.423 (12)
H19B	0.702	0.1397	0.7996	0.103*	0.423 (12)
C20B	0.573 (4)	0.277 (3)	0.7265 (18)	0.091 (7)	0.423 (12)
H20B	0.5379	0.3118	0.7658	0.109*	0.423 (12)
C21B	0.533 (6)	0.320 (5)	0.643 (3)	0.109 (7)	0.423 (12)
H21B	0.4697	0.3887	0.6272	0.13*	0.423 (12)
C22B	0.573 (5)	0.275 (4)	0.578 (5)	0.076 (6)	0.423 (12)
H22B	0.5539	0.3119	0.5207	0.091*	0.423 (12)
C23B	0.839 (4)	0.1109 (19)	0.513 (2)	0.043 (3)	0.423 (12)
H23D	0.8613	0.076	0.4714	0.065*	0.423 (12)
H23E	0.9037	0.0713	0.5571	0.065*	0.423 (12)
H23F	0.8498	0.1845	0.4849	0.065*	0.423 (12)
C24B	0.656 (6)	0.004 (4)	0.583 (5)	0.065 (8)	0.423 (12)
H24D	0.6836	−0.0266	0.5391	0.098*	0.423 (12)
H24E	0.5548	0.0119	0.5996	0.098*	0.423 (12)
H24F	0.7082	−0.0441	0.6324	0.098*	0.423 (12)
N3B	0.689 (3)	0.113 (3)	0.551 (2)	0.066 (3)	0.423 (12)
H3B	0.633	0.1554	0.507	0.079*	0.423 (12)

### Atomic displacement parameters (Å^2^)

**Table:**

	*U*^11^	*U*^22^	*U*^33^	*U*^12^	*U*^13^	*U*^23^
Sb1	0.03892 (19)	0.03206 (16)	0.03812 (18)	−0.00952 (13)	−0.00727 (14)	−0.01410 (13)
Sb2	0.0512 (2)	0.03633 (17)	0.0463 (2)	−0.01413 (15)	−0.00341 (17)	−0.01783 (15)
Br1	0.0832 (4)	0.0580 (3)	0.0507 (3)	−0.0201 (3)	−0.0150 (3)	−0.0270 (3)
Br2	0.0515 (3)	0.0522 (3)	0.0690 (4)	0.0040 (3)	−0.0174 (3)	−0.0317 (3)
Br3	0.0608 (4)	0.0566 (3)	0.0487 (3)	−0.0291 (3)	−0.0056 (3)	−0.0113 (3)
Br4	0.0485 (3)	0.0585 (3)	0.0487 (3)	−0.0159 (3)	−0.0030 (3)	−0.0185 (3)
Br5	0.0458 (3)	0.0593 (3)	0.0533 (3)	−0.0121 (3)	−0.0059 (3)	−0.0119 (3)
Br6	0.0524 (3)	0.0601 (3)	0.0475 (3)	−0.0226 (3)	−0.0070 (3)	−0.0155 (3)
Br7	0.1167 (6)	0.0462 (3)	0.0601 (4)	−0.0187 (3)	−0.0198 (4)	−0.0073 (3)
Br8	0.0693 (4)	0.0899 (5)	0.0713 (4)	−0.0423 (4)	0.0041 (3)	−0.0444 (4)
Br9	0.0767 (5)	0.0615 (4)	0.0974 (5)	−0.0210 (3)	0.0267 (4)	−0.0433 (4)
C1	0.064 (4)	0.050 (3)	0.035 (3)	−0.017 (3)	−0.011 (3)	−0.012 (2)
C2	0.064 (4)	0.073 (4)	0.051 (4)	−0.028 (3)	0.000 (3)	−0.021 (3)
C3	0.079 (5)	0.087 (5)	0.060 (4)	−0.047 (4)	−0.004 (4)	−0.019 (4)
C4	0.109 (6)	0.076 (5)	0.069 (5)	−0.036 (5)	−0.015 (5)	−0.032 (4)
C5	0.107 (7)	0.091 (5)	0.062 (5)	−0.003 (5)	−0.003 (4)	−0.042 (4)
C6	0.062 (4)	0.102 (5)	0.062 (4)	−0.022 (4)	−0.005 (3)	−0.035 (4)
C7	0.089 (5)	0.053 (3)	0.047 (3)	−0.026 (3)	−0.016 (3)	−0.011 (3)
C8	0.071 (4)	0.087 (4)	0.036 (3)	−0.028 (3)	−0.005 (3)	−0.022 (3)
N1	0.050 (3)	0.071 (3)	0.047 (3)	−0.021 (2)	−0.006 (2)	−0.020 (2)
C9A	0.034 (11)	0.091 (7)	0.062 (5)	−0.006 (7)	−0.009 (4)	−0.022 (6)
C10A	0.109 (10)	0.068 (13)	0.067 (14)	−0.033 (14)	−0.018 (10)	−0.008 (9)
C11A	0.097 (13)	0.130 (18)	0.143 (16)	−0.032 (17)	−0.027 (9)	−0.080 (19)
C12A	0.092 (9)	0.115 (16)	0.088 (9)	−0.027 (15)	−0.031 (7)	−0.032 (15)
C13A	0.055 (14)	0.089 (14)	0.113 (11)	−0.013 (10)	0.005 (8)	0.010 (11)
C14A	0.055 (11)	0.089 (8)	0.081 (7)	−0.034 (6)	−0.005 (6)	−0.048 (6)
C15A	0.055 (7)	0.056 (5)	0.099 (15)	0.001 (6)	−0.021 (8)	−0.020 (12)
C16A	0.068 (6)	0.064 (12)	0.069 (9)	−0.030 (7)	−0.007 (6)	−0.020 (7)
N2A	0.065 (7)	0.069 (4)	0.085 (5)	−0.013 (4)	−0.018 (4)	−0.033 (4)
C9B	0.034 (11)	0.091 (7)	0.062 (5)	−0.006 (7)	−0.009 (4)	−0.022 (6)
C10B	0.109 (10)	0.068 (13)	0.067 (14)	−0.033 (14)	−0.018 (10)	−0.008 (9)
C11B	0.097 (13)	0.130 (18)	0.143 (16)	−0.032 (17)	−0.027 (9)	−0.080 (19)
C12B	0.092 (9)	0.115 (16)	0.088 (9)	−0.027 (15)	−0.031 (7)	−0.032 (15)
C13B	0.055 (14)	0.089 (14)	0.113 (11)	−0.013 (10)	0.005 (8)	0.010 (11)
C14B	0.055 (11)	0.089 (8)	0.081 (7)	−0.034 (6)	−0.005 (6)	−0.048 (6)
C15B	0.055 (7)	0.056 (5)	0.099 (15)	0.001 (6)	−0.021 (8)	−0.020 (12)
C16B	0.068 (6)	0.064 (12)	0.069 (9)	−0.030 (7)	−0.007 (6)	−0.020 (7)
N2B	0.065 (7)	0.069 (4)	0.085 (5)	−0.013 (4)	−0.018 (4)	−0.033 (4)
C17A	0.054 (4)	0.067 (7)	0.038 (12)	−0.030 (4)	0.032 (7)	−0.037 (10)
C18A	0.012 (10)	0.078 (9)	0.102 (10)	0.001 (9)	−0.006 (8)	−0.054 (8)
C19A	0.062 (14)	0.145 (18)	0.073 (19)	−0.029 (10)	−0.015 (10)	−0.057 (16)
C20A	0.077 (10)	0.157 (16)	0.11 (2)	−0.042 (9)	0.030 (14)	−0.125 (19)
C21A	0.055 (17)	0.104 (13)	0.21 (3)	−0.008 (11)	−0.002 (13)	−0.116 (19)
C22A	0.047 (12)	0.082 (7)	0.116 (12)	−0.025 (8)	−0.004 (9)	−0.046 (6)
C23A	0.038 (4)	0.029 (10)	0.051 (4)	0.012 (7)	−0.001 (3)	−0.019 (8)
C24A	0.088 (15)	0.072 (9)	0.057 (13)	−0.038 (11)	0.007 (13)	−0.038 (9)
N3A	0.063 (4)	0.080 (4)	0.060 (8)	−0.015 (3)	0.012 (4)	−0.040 (5)
C17B	0.054 (4)	0.067 (7)	0.038 (12)	−0.030 (4)	0.032 (7)	−0.037 (10)
C18B	0.012 (10)	0.078 (9)	0.102 (10)	0.001 (9)	−0.006 (8)	−0.054 (8)
C19B	0.062 (14)	0.145 (18)	0.073 (19)	−0.029 (10)	−0.015 (10)	−0.057 (16)
C20B	0.077 (10)	0.157 (16)	0.11 (2)	−0.042 (9)	0.030 (14)	−0.125 (19)
C21B	0.055 (17)	0.104 (13)	0.21 (3)	−0.008 (11)	−0.002 (13)	−0.116 (19)
C22B	0.047 (12)	0.082 (7)	0.116 (12)	−0.025 (8)	−0.004 (9)	−0.046 (6)
C23B	0.038 (4)	0.029 (10)	0.051 (4)	0.012 (7)	−0.001 (3)	−0.019 (8)
C24B	0.088 (15)	0.072 (9)	0.057 (13)	−0.038 (11)	0.007 (13)	−0.038 (9)
N3B	0.063 (4)	0.080 (4)	0.060 (8)	−0.015 (3)	0.012 (4)	−0.040 (5)

### Geometric parameters (Å, º)

**Table:**

Sb1—Br1	2.6036 (6)	C11B—C12B	1.351 (11)
Sb1—Br2	2.6292 (6)	C11B—H11B	0.93
Sb1—Br3	2.6925 (6)	C12B—C13B	1.412 (11)
Sb1—Br4	3.1222 (6)	C12B—H12B	0.93
Sb1—Br5	3.0287 (6)	C13B—C14B	1.408 (10)
Sb1—Br6	2.9558 (7)	C13B—H13B	0.93
Sb2—Br4	3.1333 (7)	C14B—H14B	0.93
Sb2—Br5	3.1602 (7)	C15B—N2B	1.508 (9)
Sb2—Br6	3.2393 (7)	C15B—H15D	0.96
Sb2—Br7	2.6068 (7)	C15B—H15E	0.96
Sb2—Br8	2.5696 (7)	C15B—H15F	0.96
Sb2—Br9	2.5713 (8)	C16B—N2B	1.512 (9)
C1—C6	1.349 (8)	C16B—H16D	0.96
C1—C2	1.361 (8)	C16B—H16E	0.96
C1—N1	1.482 (7)	C16B—H16F	0.96
C2—C3	1.379 (9)	N2B—H2B	0.91
C2—H2	0.93	C17A—C18A	1.346 (14)
C3—C4	1.336 (10)	C17A—C22A	1.347 (14)
C3—H3	0.93	C17A—N3A	1.480 (9)
C4—C5	1.353 (10)	C18A—C19A	1.354 (14)
C4—H4	0.93	C18A—H18A	0.93
C5—C6	1.412 (10)	C19A—C20A	1.346 (14)
C5—H5	0.93	C19A—H19A	0.93
C6—H6	0.93	C20A—C21A	1.350 (14)
C7—N1	1.490 (7)	C20A—H20A	0.93
C7—H7A	0.96	C21A—C22A	1.352 (14)
C7—H7B	0.96	C21A—H21A	0.93
C7—H7C	0.96	C22A—H22A	0.93
C8—N1	1.495 (7)	C23A—N3A	1.482 (8)
C8—H8A	0.96	C23A—H23A	0.96
C8—H8B	0.96	C23A—H23B	0.96
C8—H8C	0.96	C23A—H23C	0.96
N1—H1	0.91	C24A—N3A	1.495 (8)
C9A—C10A	1.351 (10)	C24A—H24A	0.96
C9A—C14A	1.408 (10)	C24A—H24B	0.96
C9A—N2A	1.474 (10)	C24A—H24C	0.96
C10A—C11A	1.354 (10)	N3A—H3A	0.91
C10A—H10A	0.93	C17B—C18B	1.39 (2)
C11A—C12A	1.344 (10)	C17B—C22B	1.40 (2)
C11A—H11A	0.93	C17B—N3B	1.477 (10)
C12A—C13A	1.413 (10)	C18B—C19B	1.40 (2)
C12A—H12A	0.93	C18B—H18B	0.93
C13A—C14A	1.408 (10)	C19B—C20B	1.40 (2)
C13A—H13A	0.93	C19B—H19B	0.93
C14A—H14A	0.93	C20B—C21B	1.40 (2)
C15A—N2A	1.507 (8)	C20B—H20B	0.93
C15A—H15A	0.96	C21B—C22B	1.40 (2)
C15A—H15B	0.96	C21B—H21B	0.93
C15A—H15C	0.96	C22B—H22B	0.93
C16A—N2A	1.512 (8)	C23B—N3B	1.480 (8)
C16A—H16A	0.96	C23B—H23D	0.96
C16A—H16B	0.96	C23B—H23E	0.96
C16A—H16C	0.96	C23B—H23F	0.96
N2A—H2A	0.91	C24B—N3B	1.489 (8)
C9B—C10B	1.350 (10)	C24B—H24D	0.96
C9B—C14B	1.408 (10)	C24B—H24E	0.96
C9B—N2B	1.474 (10)	C24B—H24F	0.96
C10B—C11B	1.355 (11)	N3B—H3B	0.91
C10B—H10B	0.93		
			
Br1—Sb1—Br2	92.27 (2)	C9B—C10B—C11B	121 (3)
Br1—Sb1—Br3	91.63 (2)	C9B—C10B—H10B	119.6
Br1—Sb1—Br4	176.87 (2)	C11B—C10B—H10B	119.6
Br1—Sb1—Br5	91.05 (2)	C12B—C11B—C10B	117 (3)
Br1—Sb1—Br6	90.23 (2)	C12B—C11B—H11B	121.3
Br2—Sb1—Br3	93.37 (2)	C10B—C11B—H11B	121.3
Br2—Sb1—Br4	90.50 (2)	C11B—C12B—C13B	121 (3)
Br2—Sb1—Br5	175.26 (2)	C11B—C12B—H12B	119.3
Br2—Sb1—Br6	90.04 (2)	C13B—C12B—H12B	119.3
Br3—Sb1—Br4	89.67 (2)	C14B—C13B—C12B	122 (3)
Br3—Sb1—Br5	89.915 (19)	C14B—C13B—H13B	118.9
Br3—Sb1—Br6	176.05 (2)	C12B—C13B—H13B	118.9
Br4—Sb1—Br5	86.10 (2)	C9B—C14B—C13B	111 (2)
Br4—Sb1—Br6	88.29 (2)	C9B—C14B—H14B	124.4
Br5—Sb1—Br6	86.570 (18)	C13B—C14B—H14B	124.4
Br4—Sb2—Br5	83.71 (2)	N2B—C15B—H15D	109.5
Br4—Sb2—Br6	83.28 (2)	N2B—C15B—H15E	109.5
Br4—Sb2—Br7	172.52 (2)	H15D—C15B—H15E	109.5
Br4—Sb2—Br8	90.89 (2)	N2B—C15B—H15F	109.5
Br4—Sb2—Br9	92.67 (2)	H15D—C15B—H15F	109.5
Br5—Sb2—Br6	79.75 (2)	H15E—C15B—H15F	109.5
Br5—Sb2—Br7	91.92 (2)	N2B—C16B—H16D	109.5
Br5—Sb2—Br8	170.96 (2)	N2B—C16B—H16E	109.5
Br5—Sb2—Br9	94.81 (2)	H16D—C16B—H16E	109.5
Br6—Sb2—Br7	89.98 (2)	N2B—C16B—H16F	109.5
Br6—Sb2—Br8	92.44 (2)	H16D—C16B—H16F	109.5
Br6—Sb2—Br9	173.51 (2)	H16E—C16B—H16F	109.5
Br8—Sb2—Br9	92.68 (3)	C9B—N2B—C15B	111.9 (7)
Br8—Sb2—Br7	92.64 (3)	C9B—N2B—C16B	111.5 (7)
Br9—Sb2—Br7	93.76 (3)	C15B—N2B—C16B	110.5 (8)
C6—C1—C2	122.4 (6)	C9B—N2B—H2B	107.5
C6—C1—N1	117.5 (5)	C15B—N2B—H2B	107.5
C2—C1—N1	120.1 (5)	C16B—N2B—H2B	107.5
C1—C2—C3	117.8 (6)	C18A—C17A—C22A	117 (3)
C1—C2—H2	121.1	C18A—C17A—N3A	123 (2)
C3—C2—H2	121.1	C22A—C17A—N3A	117 (2)
C4—C3—C2	121.0 (7)	C17A—C18A—C19A	121 (2)
C4—C3—H3	119.5	C17A—C18A—H18A	119.6
C2—C3—H3	119.5	C19A—C18A—H18A	119.6
C3—C4—C5	121.7 (7)	C20A—C19A—C18A	117.8 (19)
C3—C4—H4	119.1	C20A—C19A—H19A	121.1
C5—C4—H4	119.1	C18A—C19A—H19A	121.1
C4—C5—C6	118.4 (7)	C19A—C20A—C21A	123 (2)
C4—C5—H5	120.8	C19A—C20A—H20A	118.4
C6—C5—H5	120.8	C21A—C20A—H20A	118.4
C1—C6—C5	118.7 (6)	C20A—C21A—C22A	116 (3)
C1—C6—H6	120.7	C20A—C21A—H21A	122.2
C5—C6—H6	120.7	C22A—C21A—H21A	122.2
N1—C7—H7A	109.5	C17A—C22A—C21A	123 (3)
N1—C7—H7B	109.5	C17A—C22A—H22A	118.5
H7A—C7—H7B	109.5	C21A—C22A—H22A	118.5
N1—C7—H7C	109.5	N3A—C23A—H23A	109.5
H7A—C7—H7C	109.5	N3A—C23A—H23B	109.5
H7B—C7—H7C	109.5	H23A—C23A—H23B	109.5
N1—C8—H8A	109.5	N3A—C23A—H23C	109.5
N1—C8—H8B	109.5	H23A—C23A—H23C	109.5
H8A—C8—H8B	109.5	H23B—C23A—H23C	109.5
N1—C8—H8C	109.5	N3A—C24A—H24A	109.5
H8A—C8—H8C	109.5	N3A—C24A—H24B	109.5
H8B—C8—H8C	109.5	H24A—C24A—H24B	109.5
C1—N1—C7	111.9 (4)	N3A—C24A—H24C	109.5
C1—N1—C8	113.8 (4)	H24A—C24A—H24C	109.5
C7—N1—C8	111.3 (5)	H24B—C24A—H24C	109.5
C1—N1—H1	106.5	C17A—N3A—C23A	112.7 (6)
C7—N1—H1	106.5	C17A—N3A—C24A	112.1 (6)
C8—N1—H1	106.5	C23A—N3A—C24A	112.7 (7)
C10A—C9A—C14A	119 (2)	C17A—N3A—H3A	106.2
C10A—C9A—N2A	121.4 (17)	C23A—N3A—H3A	106.2
C14A—C9A—N2A	118 (2)	C24A—N3A—H3A	106.2
C9A—C10A—C11A	120.9 (17)	C18B—C17B—C22B	125 (4)
C9A—C10A—H10A	119.5	C18B—C17B—N3B	117 (3)
C11A—C10A—H10A	119.5	C22B—C17B—N3B	111 (3)
C12A—C11A—C10A	119.2 (18)	C17B—C18B—C19B	113 (3)
C12A—C11A—H11A	120.4	C17B—C18B—H18B	123.7
C10A—C11A—H11A	120.4	C19B—C18B—H18B	123.7
C11A—C12A—C13A	124.0 (19)	C20B—C19B—C18B	124 (3)
C11A—C12A—H12A	118	C20B—C19B—H19B	117.9
C13A—C12A—H12A	118	C18B—C19B—H19B	118
C14A—C13A—C12A	114.5 (17)	C19B—C20B—C21B	112 (3)
C14A—C13A—H13A	122.8	C19B—C20B—H20B	123.9
C12A—C13A—H13A	122.8	C21B—C20B—H20B	123.9
C9A—C14A—C13A	120.6 (17)	C20B—C21B—C22B	130 (5)
C9A—C14A—H14A	119.7	C20B—C21B—H21B	115
C13A—C14A—H14A	119.7	C22B—C21B—H21B	115
N2A—C15A—H15A	109.5	C17B—C22B—C21B	108 (5)
N2A—C15A—H15B	109.5	C17B—C22B—H22B	126
H15A—C15A—H15B	109.5	C21B—C22B—H22B	126
N2A—C15A—H15C	109.5	N3B—C23B—H23D	109.5
H15A—C15A—H15C	109.5	N3B—C23B—H23E	109.5
H15B—C15A—H15C	109.5	H23D—C23B—H23E	109.5
N2A—C16A—H16A	109.5	N3B—C23B—H23F	109.5
N2A—C16A—H16B	109.5	H23D—C23B—H23F	109.5
H16A—C16A—H16B	109.5	H23E—C23B—H23F	109.5
N2A—C16A—H16C	109.5	N3B—C24B—H24D	109.5
H16A—C16A—H16C	109.5	N3B—C24B—H24E	109.5
H16B—C16A—H16C	109.5	H24D—C24B—H24E	109.5
C9A—N2A—C15A	112.1 (7)	N3B—C24B—H24F	109.5
C9A—N2A—C16A	111.6 (6)	H24D—C24B—H24F	109.5
C15A—N2A—C16A	110.5 (7)	H24E—C24B—H24F	109.5
C9A—N2A—H2A	107.5	C17B—N3B—C23B	113.1 (7)
C15A—N2A—H2A	107.5	C17B—N3B—C24B	112.6 (7)
C16A—N2A—H2A	107.5	C23B—N3B—C24B	113.4 (8)
C10B—C9B—C14B	124 (3)	C17B—N3B—H3B	105.6
C10B—C9B—N2B	124 (2)	C23B—N3B—H3B	105.6
C14B—C9B—N2B	109 (3)	C24B—N3B—H3B	105.6

### Hydrogen-bond geometry (Å, º)

**Table:**

*D*—H···*A*	*D*—H	H···*A*	*D*···*A*	*D*—H···*A*
N1—H1···Br4	0.91	2.47	3.362 (5)	168
N2*A*—H2*A*···Br3	0.91	2.43	3.23 (3)	146
N2*B*—H2*B*···Br3	0.91	2.55	3.43 (4)	162
N3*A*—H3*A*···Br6	0.91	2.4	3.25 (2)	155
N3*B*—H3*B*···Br6	0.91	2.68	3.48 (3)	148
